# Progress and Challenges in Scaling Up Laboratory Monitoring of HIV Treatment

**DOI:** 10.1371/journal.pmed.1002089

**Published:** 2016-08-23

**Authors:** Peter H. Kilmarx, Raiva Simbi

**Affiliations:** 1 Fogarty International Center, National Institutes of Health, Bethesda, Maryland, United States of America; 2 Ministry of Health and Child Care, Harare, Zimbabwe

## Abstract

In a perspective on Habiyambere and colleagues, Peter Kilmarx and Raiva Simbi discuss the disconnect between HIV testing instrument capacity and utilization.

UNAIDS has set ambitious global targets for the year 2020 of diagnosing 90% of all people living with HIV (PLWHIV), initiating antiretroviral treatment (ART) for 90% of those diagnosed with HIV infection and achieving an undetectable viral load in 90% of those on ART [1]. Significant progress has been made in reaching that goal. As of the end of 2015, 17 million people were accessing ART, which was over 45% of all PLWHIV [2].

Laboratory testing is a critical component in reaching these goals [3]. Measurement of CD4+ T-lymphocytes (CD4 testing) is used to assess immunologic suppression from HIV infection and response to ART, while viral load testing is used to assess ART efficacy. The World Health Organization (WHO) guidelines call for CD4 testing every six months and for viral load testing after six months and every 12 months thereafter for patients on ART [4]. New WHO HIV treatment guidelines, which recommend initiation of ART at any CD4 count, emphasize the importance of viral load relative to CD4 testing [5].

In this issue, Habiyambere and colleagues report the results of annual global surveys on availability and use of these testing technologies [6]. Questionnaires were administered through WHO country offices to national HIV program managers in 127 countries, focused on countries with the greatest burden of HIV and AIDS. The results indicate that, worldwide, there was a surplus of testing instrument capacity to conduct CD4 testing, enough for 4.6 tests per year for every PLWHIV and 12.8 per year for every person on ART in 2013, far more tests than what is recommended. However, only 1.4 tests per person on ART were conducted, with a capacity utilization rate of only 13.7%. For viral load testing, in 2013 there was sufficient testing instrument capacity for 0.4 tests per PLWHIV and 1.2 tests per person on ART, but only 0.5 tests per person on ART were conducted, with a capacity utilization rate of only 36.5%. Testing coverage rates and testing capacity utilization rates varied by region. The results for CD4 testing were similar in Africa, which bears the greatest burden of HIV and AIDS, compared to the global figures, while for viral load there was lower testing instrument capacity per person on ART but greater utilization rates in Africa.

Why is there such a striking disconnect between capacity and utilization? The survey found that lack of machine installation, breakdowns, and lack of reagents were all factors, and most machines were not covered by maintenance contracts or receiving recommended service [4]. But underlying these explanations are deeper challenges in management of a remarkably complex system. The correct equipment must be procured and deployed appropriately to meet the catchment area need. Patient and specimen numbers must be forecast correctly. Specimen transport systems that ensure specimen integrity must be developed, often de novo for HIV programs in resource-poor settings. Reagents must be procured and distributed in the right quantity to have sufficient stock while avoiding expiration. Laboratory technicians must be trained and retained in service, and machines must be properly maintained. The WHO 2014 updated technical and operational considerations for implementing HIV viral load testing provide helpful guidance for further scaling up [7].

The results suggest that some programs may have overly focused on machine procurement and did not plan appropriately for the other critical aspects of a functioning national HIV laboratory monitoring system. Funding organizations must commit to supporting the full spectrum of laboratory program needs. Laboratory directors need the program management training, tools, resources, and authorities for implementation. Ensuring transparent public procurement of these complex technical products and services is also critical [8]. Use of unique national technical specifications and participation of shell corporations are special risk factors for corruption in public procurements. Minimum standards and checklists have been developed to counter corruption; these include a recommendation that civil society be engaged in monitoring of public procurements [9].

While Habiyambere et al. have highlighted critical shortfalls in CD4 and viral load testing, the problem is even greater when considering the gaps in the entire cascade of events, which are not captured in their survey, starting with clinicians ordering tests when appropriately indicated and ending with timely return of results and using them for appropriate clinical decision making [10]. These gaps are manifest in early infant diagnostic testing. The results from Habiyambere et al. indicate that infants are being tested, with 1.2 tests conducted per HIV-exposed infant [4], yet pediatric ART coverage lags far behind adult treatment, with only half of HIV-infected children worldwide on ART in 2013 [11]. The stark lack of machine maintenance as well as challenges with specimen quality also raises concerns about test accuracy. Implementation of comprehensive laboratory quality assurance programs and accreditation, which include proficiency testing, must be prioritized in HIV laboratory support [12].

Much has been accomplished to date, even in settings with limited laboratory capacity. In Zimbabwe, for example, nearly 900,000 people have been initiated on ART with good levels of retention and adherence by global standards, and the great majority of patients are doing well on first-line therapy [13]. Scaling up viral load testing was carefully planned with a phased approach [14], as recommended [5]. However, despite a goal of 21% coverage of ART patients with targeted viral load testing in 2015, only 5.6% was achieved, owing largely to challenges with resource mobilization, equipment procurement, and specimen transport.

A sort of “honeymoon” period in HIV treatment in resource-poor settings, when most patients were treatment naïve, initially symptomatic, and highly adherent, and high levels of viral load suppression could be confirmed with periodic representative samples of ART patients, is coming to an end as both transmitted resistance and acquired resistance are increasing worldwide [15]. In this new era, individual patient viral load monitoring will be critical to ensure treatment response, judicious use of costly second- and third-line antiretroviral medications, and minimal development and spread of resistance. Strong leadership, resources, planning, and management are needed to scale up laboratory services. Continuing monitoring efforts, like those of Habiyambere and colleagues, are essential.

## Abbreviations

ART: antiretroviral treatment
PLWHIV: people living with HIV
